# Microbial Associations of Abyssal Gorgonians and Anemones (>4,000 m Depth) at the Clarion-Clipperton Fracture Zone

**DOI:** 10.3389/fmicb.2022.828469

**Published:** 2022-03-30

**Authors:** Elena Quintanilla, Clara F. Rodrigues, Isabel Henriques, Ana Hilário

**Affiliations:** ^1^Centre for Environmental and Marine Studies (CESAM), Department of Biology, University of Aveiro, Aveiro, Portugal; ^2^Department of Life Sciences, Faculty of Science and Technology, University of Coimbra, Coimbra, Portugal

**Keywords:** microbiome, abyssal, deep-sea corals, deep-sea anemones, Clarion-Clipperton Fracture Zone (CCZ), polymetallic nodules

## Abstract

Deep coral-dominated communities play paramount roles in benthic environments by increasing their complexity and biodiversity. Coral-associated microbes are crucial to maintain fitness and homeostasis at the holobiont level. However, deep-sea coral biology and their associated microbiomes remain largely understudied, and less from remote and abyssal environments such as those in the Clarion-Clipperton Fracture Zone (CCZ) in the tropical Northeast (NE) Pacific Ocean. Here, we study microbial-associated communities of abyssal gorgonian corals and anemones (>4,000 m depth) in the CCZ; an area harboring the largest known global reserve of polymetallic nodules that are commercially interesting for the deep-sea nodule mining. Coral samples (*n* = 25) belonged to Isididae and Primnoidae families, while anemones (*n* = 4) to Actinostolidae family. Significant differences in bacterial community compositions were obtained between these three families, despite sharing similar habitats. Anemones harbored bacterial microbiomes composed mainly of Hyphomicrobiaceae, Parvibaculales, and *Pelagibius* members. Core microbiomes of corals were mainly dominated by different Spongiibacteraceae and Terasakiellaceae bacterial members, depending on corals’ taxonomy. Moreover, the predicted functional profiling suggests that deep-sea corals harbor bacterial communities that allow obtaining additional energy due to the scarce availability of nutrients. This study presents the first report of microbiomes associated with abyssal gorgonians and anemones and will serve as baseline data and crucial insights to evaluate and provide guidance on the impacts of deep-sea mining on these key abyssal communities.

## Introduction

Deep-water coral communities are found distributed from Arctic to Antarctic latitudes, along continental margins and on seamounts (Roberts et al., 2006; Yesson et al., 2012). As well as their shallow-water counterparts, deep-sea corals provide substrate and habitat for many associated biota, increasing biomass and biodiversity of benthic communities (Buhl-Mortensen and Mortensen, 2005). They are considered engineering species that shape the habitat and increase its complexity by forming three-dimensional structures (Jones et al., 1994; Ballesteros, 2006; Sánchez, 2016). Moreover, corals play key roles in biogeochemical cycles. Thanks to their condition of filter feeders, corals are responsible for benthic-pelagic coupling processes by promoting the flow of matter and energy from pelagic to benthic systems (Gili and Coma, 1998; Ribes et al., 1999; Hill et al., 2014).

Due to the limited access to surface irradiance, deep-sea corals lack photosynthetic symbionts. In turn, they obtain nutrients heterotrophically by filter feeding but also from their associated microbiomes (White et al., 2005; Zhang et al., 2015). The microbiome is the microbial community comprising protists, bacteria, archaea, viruses and fungi that are hosted in the coral holobiont (Rohwer et al., 2002). It provides crucial benefits for maintaining the coral holobiont health and dynamics by their involvement in nutrient cycling, key metabolic pathways and disease resistance (Rosenberg et al., 2007; Rypien et al., 2010; Zhang et al., 2015). Therefore, the microbiome augments basic life functions to the coral holobiont but at the same time contributes to adaptive and acclimatization responses by increasing fitness and maintaining homeostasis at the holobiont level (Bourne et al., 2016; Ziegler et al., 2017, 2019; Marangon et al., 2021).

Unfortunately, deep-sea coral biology and ecology remain vastly understudied due to logistical constraints and sampling challenges and consequently, few studies have addressed deep-sea coral microbiomes. Of the best studied are the deep-sea stony corals *Desmophyllum pertusum* (formerly *Lophelia pertusa*) and *Madrepora oculata* (Yakimov et al., 2006; Neulinger et al., 2008; Schöttner et al., 2009; Kellogg et al., 2017; Galand et al., 2018). However, few microbial studies have evaluated deep-sea octocorals including *Paragorgea arborea*, *Plumarella superba*, *Cryogorgia koolsae*, *Paramuricea placomus*, *Primnoa* spp., *Anthothela* spp., and *Acanthogorgia* spp. (Gray et al., 2011; Kellogg et al., 2016; Lawler et al., 2016; Goldsmith et al., 2018; Weiler et al., 2018; Vohsen et al., 2019; Kellogg and Pratte, 2021). Moreover, none of these studies comprised specimens living below 1,600 m depth and, to our knowledge, no studies exist addressing microbial associates of deep-sea anemones.

Deep-sea corals and anemones are also found in abyssal zones (i.e., at depths between 3,000 and 6,000 m). One example is the abyssal sea floor of the Clarion-Clipperton Fracture Zone (CCZ) in the tropical Northeast (NE) Pacific Ocean. This is a vast and highly remote area harboring the largest known global reserve of polymetallic nodules, which are commercially interesting for the seafloor mining (Ramirez-Llodra et al., 2011). The CCZ is undergoing intense exploration for potential nodule extraction with currently 17 exploration contracts issued by the International Seabed Authority. Surprisingly, high levels of diversity have been associated to the abyssal seafloor (De Smet et al., 2017; Wilson, 2017; Hauquier et al., 2018). Specifically, abyssal epifauna including ophiuroids, sponges, echinoderms, hydrozoans, corals, and anemones live associated to these polymetallic deposits (Amon et al., 2016; Christodoulou et al., 2020). Therefore, mining the nodules would cause an unprecedented impact on the abyssal communities, making the conservation of CCZ biodiversity of critical importance (Weaver et al., 2018). For instance, sediment plumes generated by these anthropogenic activities may bury benthic organisms preventing larval settlement and colonization and ultimately triggering habitat and biodiversity loss (Ramirez-Llodra et al., 2011; Vanreusel et al., 2016; Stratmann et al., 2018). Particularly, microbiome disruptions following nodule mining may have direct impacts on coral’s viability and resilience, generating severe ecological effects at the community level and thus impacting one of the most remote and least studied environments on the planet (Wear et al., 2021).

In order to assess and predict direct and indirect impacts of polymetallic nodule mining on these benthic communities, it is crucial to obtain baseline data prior to exploitation. Accordingly, in this study we identify for the first time the bacterial microbiome of abyssal corals and anemones from the CCZ. Specifically, we address bacterial community composition and evaluate the predicted functional diversity of the microbiome within the holobiont in organisms found deeper than 4,000 m depth. These valuable results are crucial to understand responses and evaluate the resilience capacity of key benthic organisms at the holobiont level when facing local disturbances. Ultimately, our results will serve as essential insights to assess and provide guidance on the impacts of deep-sea mining on these crucial benthic communities.

## Materials and Methods

### Study Site and Sample Collection

**FIGURE 1:**
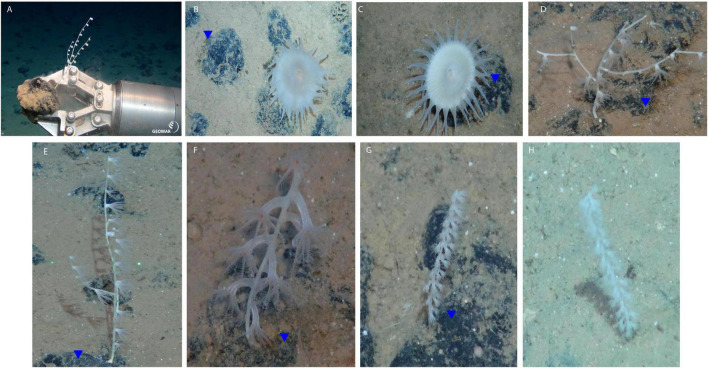
Sample collection and specimens. **(A)** ROV Kiel 6000 GEOMAR manipulator arm collecting a coral sample attached to a polymetallic nodule. **(B,C)** Actinostolidae, **(D–F)** Isididae, **(G,H)** *Calyptrophora* sp. (Primnoide). Note that blue triangles point polymetallic nodules. Photo credits: GEOMAR.

Specimens were collected during the SO268 cruise on board RV Sonne (18 February – 21 May 2019) in the CCZ, in the tropical northeast Pacific Ocean, at depths varying from 4,089 to 4,543 m (see Table 1). Coral and anemones samples came from two contract areas: the central GSR area (G-TEC Sea Mineral Resources NV, Belgium) and the eastern most BGR area (Federal Institute for Geosciences and Natural Resources, Germany). Samples were collected with the manipulator arm (see Figure 1) of the remotely operated vehicle (ROV) Kiel 6000 (GEOMAR) and placed in separated thermally stable containers that were sealed at depth to prevent microbial contamination from other specimens or different water masses during ascent. Each container was washed, ethanol sterilized, filled with freshwater, and sealed while the ROV was on deck. The freshwater evacuated at depth when the container was opened to receive the coral sample, so that only seawater local to the coral samples was entrained during collection. Once on deck the containers were immediately transferred to a cold room (4–7°C) for sample processing.

**TABLE 1:** Summary of specimens’ taxonomy, collection sites, and depth.

Specimens	Longitude	Latitude	Depth (m)	Contract area	18S rRNA barcoding result	Blast similarity (%)
A72	−125.869954	14.111864	4,506	GSR	Actinostolidae	99.34
A149	−125.871286	14.110831	4,496	GSR	Actinostolidae	99.34
A155	−125.924636	14.033209	4,543	GSR	Actinostolidae	99.57
A159	−125.923382	14.033285	4,543	GSR	Actinostolidae	99.46
C1	−117.024506	11.929556	4,085	BGR	Isididae	99.65
C05	−117.024036	11.928.241	4,089	BGR	Isididae	99.31
C10	−117.024051	11.92825	4,089	BGR	Isididae	99.03
C11	−117.024079	11.928231	4,089	BGR	Isididae	99.09
C100	−117.012954	11.861419	4,130	BGR	Isididae	100
C101	−117.012724	11.860827	4,129	BGR	Isididae	100
C102	−117.012821	11.861526	4,130	BGR	Isididae	100
C94	−117.012815	11.862358	4,128	BGR	*Calyptrophora* sp.	99.46
C95	−117.012883	11.862227	4,129	BGR	*Calyptrophora* sp.	99.78
C96	−117.012865	11.862183	4,129	BGR	*Calyptrophora* sp.	99
C97	−117.012922	11.861812	4,130	BGR	*Calyptrophora* sp.	98
C99	−117.01298	11.861698	4,130	BGR	Isididae	98.37
C182	−117.023986	11.928615	4,090	BGR	Isididae	99.11
C183	−117.024023	11.928651	4,090	BGR	Isididae	100
C184	−117.024013	11.928645	4,090	BGR	*Calyptrophora* sp.	97.24
C185	−117.024023	11.928694	4,090	BGR	Isididae	99.89
C186	−117.023969	11.929118	4,089	BGR	Isididae	99.87
C187	−117.024139	11.928336	4,090	BGR	Isididae	99.10
C247	−117.012868	11.86288	4,127	BGR	Isididae	98.49
C249	−117.012864	11.862905	4,127	BGR	Isididae	93.20
C250	−117.012774	11.862914	4,127	BGR	Isididae	99.23
C251	−117.012805	11.862912	4,127	BGR	*Calyptrophora* sp.	98.51
C252	−117.012719	11.862925	4,127	BGR	*Calyptrophora* sp.	97.49
C253	−117.012635	11.862949	4,127	BGR	Isididae	99.28
C254	−117.012624	11.862928	4,127	BGR	Isididae	99.77

*GSR: central area (G-TEC Sea Mineral Resources NV, Belgium). BGR: eastern most area (Federal Institute for Geosciences and Natural Resources, Germany).*

From each colony and individual specimens, two samples were collected of approximately 5 cm length. One sample was stored in alcohol 96% for molecular taxonomic identification. The other sample was stored in RNAlater (Thermo Fisher Scientific, Waltham, MA, United States) and preserved at −80°C until subsequent microbiome DNA extraction. Prior to DNA extractions, all samples were gently rinsed with 100 ml filtered fresh water in order to remove exogenous or transient microorganisms loosely associated with the coral or anemone tissues.

### Taxonomic Assignment

Cnidarian specimens were identified based on the sequences of their 18S rRNA genes. DNA was extracted using the ISOLATE II Genomic DNA Kit (BIOLINE). Fragments of the nuclear loci encoding 18S rRNA were amplified. Approximately 1,600 bp of 18S was amplified using the primers 18SA (AYCTGGTTGATCCTGCCAGT) and 18SB (ACCTTGTTACGACTTTTACTTCCTC). PCR mixtures contained 1 μl of each primer (10 μM), 1 μl template DNA and 12.5 μl of NZYTaq II 2× Green Master Mix (NYZTECH) in a mixture of total 25 μl. The PCR amplification profile consisted of initial denaturation at 95°C for 5 min, 35 cycles of denaturation at 94°C for 45 s, annealing at 55°C for 45 s, extension at 72°C for 2 min, and a final extension at 72°C for 10 min following (Dahlgren et al., 2016). PCR products were purified using the ISOLATE II PCR and Gel Kit (BIOLINE) and sent for sequencing in both directions at Eurofins Genomics (Germany). Overlapping sequence fragments were merged into consensus sequences and aligned using BIOEDIT 7.3 (Hall, 1999). DNA sequences obtained were identified using BLAST^1^ (Altschul et al., 1997).

### Microbiome DNA Extraction and 16S rRNA Gene Sequencing

DNA was extracted using the DNeasy Blood & Tissue Kit (Qiagen) after macerating each sample in liquid nitrogen. DNA was quantified using a Nanodrop 2000 UV-Bis Spectrophotometer (Thermo Fisher Scientific). Reagent-only controls were included for extraction kit and PCR to identify reagent contamination (Salter et al., 2014; Pollock J. et al., 2018). Samples were processed in multiple batches (i.e., two batches of 10 samples and 1 batch of 9 samples, which were processed in different days). The variable V3–V5 region of the 16S rRNA gene was sequenced using the 341F (5′-CCTACGGGNGGCWGCAG-3′, Herlemann et al., 2011) / 926R (5′-GGGTTGCGCTCGTTGCGGG-3′, Sacchi et al., 2002) PCR primers and Illumina flowcell adapter sequences. Barcoded amplicons were pooled and sequenced on the Illumina MiSeq platform (Eurofins Genomics, Germany), implementing 2 × 300 bp paired-end read libraries.

### Bioinformatics and Statistical Analyses

Amplicon sequence data were demultiplexed, assembled and analyzed using QIIME2 (v2021.4) (Bolyen et al., 2019) to identify Amplicon Sequence Variants (ASVs). Denoising, chimera filtering, and trimming was performed with DADA2 (Callahan et al., 2016). Since the resulting sequenced amplicons were too large for an overlap, we finally had to use single-end data (i.e., forward sequences) with a final amplicon length of 300 bp for subsequent analyses. Microbial taxonomy was assigned using a naive Bayes classifier trained on the SILVA 132 99% database (silva-132-99-nb-classifer). Singleton ASVs were removed to minimize false ASVs, as well as ASVs identified as eukaryotes, mitochondria or chloroplasts. The sequences were deposited in the NCBI Sequence Read Archive (SRA) under BioProject number PRJNA784368.

Diversity indices of the samples were assessed using the QIIME 2 pipeline.^2^ Alpha diversity indices (total number of observed ASVs, Shannon diversity and Faith’s phylogenetic diversity) were assessed to determine the microbial diversity within each sample (Shannon, 1948; Faith, 1992). Pairwise comparisons of alpha diversity metrics between taxonomic groups were made using non-parametric Kruskal–Wallis tests. Multivariate analyses were conducted to assess differences in bacterial community compositions between samples (beta diversity). In order to visualize differences in bacterial community composition between samples we performed principal coordinate analyses (PCoA), applying a square-root transformation to relative abundances and calculating Bray–Curtis dissimilarity matrices. Permutational Analyses of Variance (PERMANOVA; Anderson, 2001) were conducted to test differences in bacterial community composition between samples (9,999 permutations). Similarity Percentage (SIMPER) analyses were used to identify the taxa contributing to the greatest extent to the observed patterns. Additionally, heatmap was generated with the R package ggplot2 (Wickham, 2009) through the RStudio suite to visualize patterns of similarity in ASVs’ abundances between samples.

### Taxonomy-Based Functional Profiling

Putative functional differences inferred from differences in bacterial community composition among samples were assessed by using METAGENassist (Arndt et al., 2012). ASVs filtering and normalization parameters were used as described by Hadaidi et al. (2017). Euclidean distance measure (single linkage algorithm) was used to visualize functional profiles (i.e., metabolism, oxygen requirements, carbon, and energy source) in heatmaps mapped to the microbial communities.

## Results

Taxonomy of samples was DNA-based identified. Coral specimens belonged to Primnoidae (*Calyptrophora* sp.) and Isididae families (Alcyonacea, Calcaxonia), while anemone specimens belonged to Actinostolidae family (Actinaria, Enthemonae) (Figure 1). Taxonomy results based on 18S rRNA barcoding are shown in Table 1 and the respective GenBank accession numbers are comprised between OM522904 and OM522932.

**FIGURE 2:**
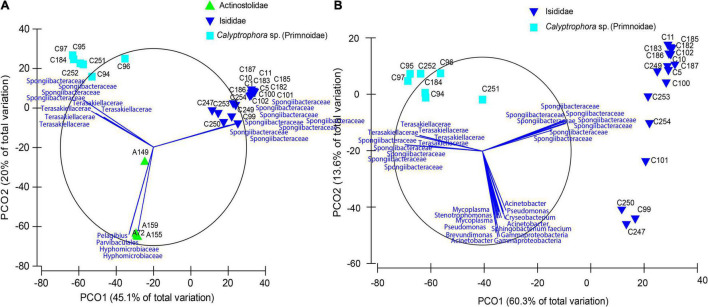
Principal coordinate analyses plots based on a Bray–Curtis dissimilarity matrix of bacterial community compositions in anemones and coral samples. **(A)** Community compositions from samples belonging to Primnoidae (*Calyptrophora* sp.), Isididae, and Actinostolidae families and **(B)** from coral samples belonging to Isididae and Primnoidae (*Calyptrophora* sp.). Bray–Curtis dissimilarity metrics on the square-root transformed relative abundances were used to compare samples. Principal coordinate analysis was used for visualization purposes, and the first two components (explaining over 50% of the variation) are displayed. Vectors correspond to taxa mainly conducting sample differentiation.

**FIGURE 3:**
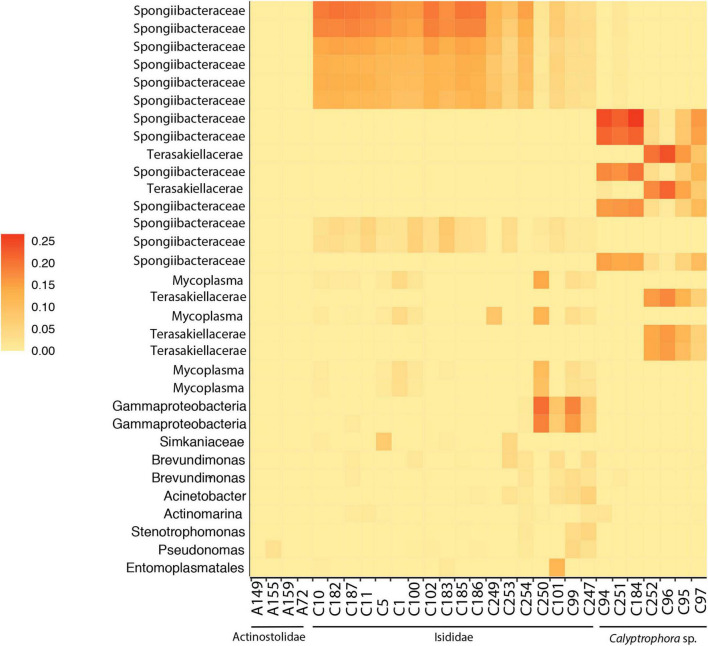
Heatmap of the relative abundances of ASVs taxa accounting for the cumulative 80% of total abundance in anemones (A) and coral (C) samples.

The final dataset consisted of 29 individual samples. After filtering, a total of 88 ASVs per sample were obtained together with a mean of 14,617 reads per sample. Rarefaction curves for all samples plateaued before 5,000 sequencing depth, indicating a good representation of bacterial diversity (Supplementary Figure 1). Significant differences in observed ASVs and Faith’s phylogenetic diversity were obtained between Actinostolidae and Primnoidae samples (*P* = 0.041 and *P* = 0.0415, respectively). Additionally, significant differences in Shannon diversity were obtained between Actinostolidae and Isididae (*P* = 0.034) and between Actinostolidae and Primnoidae (*P* = 0.027). Beta diversity measurements visualized by principal coordinate analysis revealed sample clustering in three well-differentiated groups corresponding to the three taxonomic families (i.e., Primnoidae, Isididae, and Actinostolidae; Figure 2A). Interestingly, within Actinostolidae samples, a clear differentiation in microbiome composition is observed for A149 from the rest of samples (Figure 2A). Significant differences in community compositions were inferred between these three sampling groups (PERMANOVA, *P* < 0.05, Permutational Analyses of Multivariate Dispersions, Table 2). ASVs mainly driving the differentiation between the three sampling groups were taxonomically assigned to Spongiibacteraceae (Cellvibrionales, Gammaproteobacteria) for Isididae corals, Spongiibacteraceae, and Terasakiellaceae (Rhodospirillales, Alphaproteobacteria) for Primnoidae corals, and Hyphomicrobiaceae, Parvibaculales, and *Pelagibius* for the anemones (Figures 2A, 3 and Supplementary Figure 2). SIMPER analyses revealed 86.84% of dissimilarity between anemones and Isididae corals, 99.11% of dissimilarity between anemones and Primnoidae corals and 93.50% of dissimilarity between Isididae and Primnoidae corals (Supplementary Figure 2).

**TABLE 2:** PERMANOVA analyses.

PERMANOVA pair-wise tests		
**Groups**	***t***	***P* (perm)**
A, C2	3.84	0.0002*
A, C1	3.26	0.0032*
C1, C2	5.34	0.0001*

Anemones (A), Calyptrophora sp. (Primnoidae) (C1), and Isididae (C2). *P < 0.05.

Principal coordinate analysis revealed a gradient of variation in the microbial community in Isididae and Primnoidae corals, being able to identify different potential groups within each coral family (Figures 2B, 3). Different ASVs mainly corresponding to Spongiibacteraceae and Terasakiellaceae drove the differentiation between the group of samples from Primnoidae (*Calyptrophora* sp.) and Isididae corals (Figures 2B, 3 and Supplementary Figure 2). Additionally, some Isididae samples showed moderate abundant ASVs belonging to *Mycoplasma*, *Brevundimonas*, and *Acinetobacter* bacteria (Figure 3). No significant differences in bacterial community compositions were obtained between the two defined sampling areas, according to the sampling coordinates (PERMANOVA, *P* = 0.102).

**FIGURE 4:**
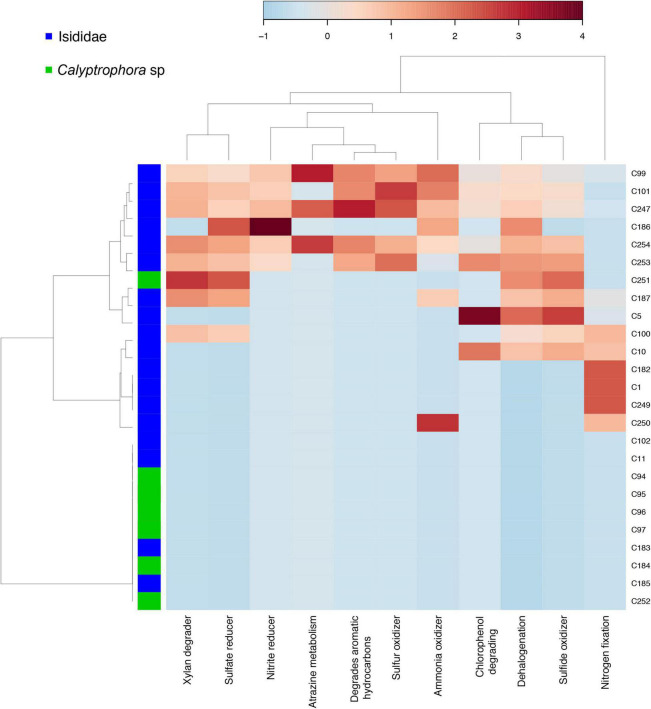
Taxonomy-based functional profiling of bacterial communities in coral samples from Isididae and Primnoidae families. Shifts in potential functional differences are represented by a relative abundance scale showing the enrichment (red color) and depletion (blue color) in different metabolic profiles mapped to the corresponding taxonomic information. Hierarchical clustering of samples and functions was performed by a single linkage algorithm using Euclidean distance measurements.

In addition to differences in bacterial community composition, we also observed differences in taxonomy-based functional profiles mainly between Isididae and Primnoidae coral families. Specifically, some Isididae individuals showed enriched bacterial taxa related to metabolic categories such as “xylan degrader,” “sulfate reducer,” “nitrite reducer,” “sulfur and ammonia oxidizer,” “dehalogenation,” “sulfide oxidizer” and “nitrogen fixation,” “chlorophenol degrading” and “aromatic hydrocarbons” (Figure 4).

## Discussion

We report for the first time the microbiome structure of polymetallic nodule-associated gorgonian corals and anemones in the abyssal CCZ (>4,000 m depth). Our results revealed evident bacterial community differences between corals and anemones sharing similar habitats. Additionally, differences in microbial composition between specimens from Primnoidae and Isididae coral families were driven mainly by different ASVs belonging to Spongiibacteraceae and Terasakiellaceae.

Bacterial microbiomes were significantly different between anemones and gorgonian corals. Although the sampled animals shared similar habitat conditions (i.e., depth and benthic communities), bacterial compositions totally differed depending on the type of the cnidarian host. This is in line with the host-microbe coevolution concept between host linages and their symbiont microbial communities (Pollock F. J. et al., 2018; O’Brien et al., 2019). Since microbiomes are vital to maintain homeostasis, the holobiont can have selected beneficial symbionts to establish specific and strong relationships over time (Bordenstein and Theis, 2015), potentially explaining the differentiation between microbiomes hosted by corals and anemones. Additionally, polyps size differentiation and thus, diet variation, may also contribute to differences in bacterial microbiomes between different benthic organisms (Galand et al., 2020).

Microbiome of Actinostolidae anemones were composed mainly of bacteria belonging to Parvibaculales, *Pelagibius* (Rhodovibrionales, Kiloniellaceae) and Hyphomicrobiaceae (Rhizobiales). The order Parvibaculales has been recently found as dominant microbiome members of coral reef sponges (Baquiran et al., 2020; Robbins et al., 2021) and have been associated to temporal dynamics (seasonal changes) (Mena et al., 2020; Deutschmann et al., 2021). Additionally, these bacteria have been positively correlated with ammonia concentrations in the water column (Muck et al., 2019). Members of *Pelagibius* genera are strict aerobic bacteria that have been found in coastal waters (Choi et al., 2009) but also belong to the core microbiome of the temperate coral *Oculina patagonica* (Rubio-Portillo et al., 2016). Members of *Pelagibius* and Hyphomicrobiaceae were isolated from lobster lesions, suggesting their role as opportunistic colonizers (Quinn et al., 2012). This taxon is involved in nutrient cycling including denitrification and has been found among the most abundant bacteria in polymetallic nodules, being potential metal-cycling bacteria (Cleary et al., 2015; Molari et al., 2020). Most of Hyphomicrobiaceae bacteria are aerobic chemoheterotrophs and oligocarbophilic, thriving only in the presence of low concentrations of suitable carbon sources (Oren and Xu, 2014). These findings suggest that these deep-sea cnidarians establish symbiotic associations with chemosynthetic bacteria as an alternative nutritional strategy from conventional sea anemones (i.e., strict suspension feeders or prey capture animals).

Isididae and Primnoidae gorgonian corals showed evident differences in their microbial communities. Interestingly, these differences were mainly driven by ASVs from the same taxonomic classification: Terasakiellaceae and Spongiibacteraceae bacteria. Different Terasakiellaceae ASVs were associated to Primnoidae corals. These bacterial taxa have been found associated to marine sponges, scleractinian corals and to the deep-sea gorgonian *Paragorgia arborea* (Weiler et al., 2018; Sacristán-Soriano et al., 2019; Parker et al., 2020). It is suggested that bacteria belonging to Terasakiellaceae family are involved in nitrogen cycling, especially in habitats where food limitation is noticeable, as the deep-sea (Weiler et al., 2018). Interestingly, the most abundant bacteria found in Isididae and Primnoidae corals belonged to Spongiibacteraceae family. However, these two coral families did not share any ASVs from this bacteria family. Spongiibacteraceae is typically found in coral holobionts as have been associated to scleractinian corals including *Acropora* and *Coelastrea* species and the deep-water coral *D. pertusum* (Gardner et al., 2019; Jensen et al., 2019). Members of Spongiibacteraceae are known as hydrocarbons and steroid-degrading bacteria for harvesting additional energy (Holert et al., 2018; Dhal et al., 2020). Moreover, some Isididae samples showed four moderately abundant ASVs from *Mycoplasma.* This bacterial group has been found associated to Isididae corals from Alaska (Penn et al., 2006). Additionally, *Mycoplasma* were found associated to deep-sea corals and gorgonians including *D. pertusum*, *C. koolsae* and *P. superba*, *Pacifigorgia cairnsi* and *P. placomus* (Neulinger et al., 2009; Gray et al., 2011; Kellogg et al., 2016; Quintanilla et al., 2018). Although *Mycoplasma* have been considered endosymbionts in corals and in some cnidarian, their specific role within the coral holobiont remains unclear (Neulinger et al., 2009; Weiland-Bräuer et al., 2015).

Remarkably, none of the coral samples include *Endozoicomonas* in their core microbiomes. This pattern is concordant with microbiomes of the deep-sea coral *D. pertusum* and the deep-sea gorgonians *P. placomus* and *Anthothela grandiflora* (van Bleijswijk et al., 2015; Kellogg et al., 2016; Lawler et al., 2016). However, microbiomes of deep-sea *Acanthogorgia* spp. and *D. pertusum* have been recently reported to be dominated by this bacterial group (Kellogg and Pratte, 2021). *Endozoicomonas* is a dominant genus in temperate and tropical corals that play important roles in coral’s health by providing antimicrobial activity, and being involved in multiple metabolic functions (Ding et al., 2016; Neave et al., 2017). Although coral host selecting for or against *Endozoicomonas* may be contributing to explain this pattern (Meistertzheim et al., 2016; Galand et al., 2018; Chapron et al., 2020), it is also probable that specific environment conditions such as depth, pressure, temperature and availability of food resources relate with the absence of *Endozoicomonas* members in our Isididae and Primnoidae corals. This raises an interesting question as whether highly abundant Spongiibacteraceae members present in our Isididae and Primnoidae samples are playing equivalent roles as *Endozoicomonas* in tropical and temperate shallow gorgonians in order to maintain holobiont homeostasis.

Some Isididae samples showed bacterial taxa with the potential to fix nitrogen and to use organic carbon resources like xylan. Since deep-sea corals depend on heterotrophy, association with bacteria that can fix nutrients is crucial to cover the holobiont demands (Middelburg et al., 2015; Röthig et al., 2017). Specifically, sulfate and nitrite reducing bacteria may indicate the capacity of some microbiome components to use other elements as distinct functional adaptations in challenging environments (Lawler et al., 2016; Röthig et al., 2017). Then, evidence of specific metabolic pathways such as those belonging to nitrogen cycling suggest nutrition supplement in cold-water corals (Middelburg et al., 2015). Moreover, the presence of sulfur-oxidizing bacteria in Isididae samples may correspond to the availability of nitrogen compounds derived from organic matter decomposition (Hensen et al., 2006). Interestingly, the most abundant ASVs found in our samples, Spongiibacteraceae, are considered marine hydrocarbon-degrading bacteria with the potential to harvest additional energy (Dhal et al., 2020). Therefore, the predicted functional profiling found in our results suggest that deep-sea corals harbor bacterial communities that allow obtaining and using additional energy due to the scarcity of nutrients at such depths.

Finally, it is worthy to point out that differences in microbiome compositions were also observed within anemones and within each of the two coral families. Differences in host-associated microbiomes among cnidarians from low or same taxonomic levels may be driven by host-specificity, host health status and/or environmental settings (van de Water et al., 2016, 2017; Brown et al., 2017; Pollock F. J. et al., 2018; Quintanilla et al., 2018). Undoubtedly, higher taxonomic resolution of samples would help to interpret these results.

Our study reveals the bacterial microbiome composition of gorgonian corals and anemones from the abyssal sea floor of the CCZ in the tropical NE Pacific Ocean. The study recognizes specific microbiome compositions according to the host taxonomy, despite sharing similar habitats. Given the pivotal role that microbiome plays in holobiont health status, future studies should focus on elucidating direct and indirect impacts of deep-sea nodule mining on microbiome disruptions and resilience capacity of these key benthic communities.

## Data Availability Statement

The datasets presented in this study can be found in online repositories. The names of the repository/repositories and accession number(s) can be found in the article/Supplementary Material.

## Author Contributions

EQ, IH, and AH contributed to the design of the study. AH conducted the sampling. EQ and CR generated the data. EQ analyzed the data and wrote the manuscript. EQ, CR, IH, and AH interpreted the data results and revised the manuscript. All authors contributed to the article and approved the submitted version.

## Conflict of Interest

The authors declare that the research was conducted in the absence of any commercial or financial relationships that could be construed as a potential conflict of interest.

## Publisher’s Note

All claims expressed in this article are solely those of the authors and do not necessarily represent those of their affiliated organizations, or those of the publisher, the editors and the reviewers. Any product that may be evaluated in this article, or claim that may be made by its manufacturer, is not guaranteed or endorsed by the publisher.

## Footnotes

1
http://www.ncbi.nlm.nih.gov

2
https://docs.qiime2.org
