# Supplementary material for: Microbial Associations of Abyssal Gorgonians and Anemones (>4,000 m Depth) at the Clarion-Clipperton Fracture Zone
Source: Front Microbiol. 2022 Mar 30;13:828469. doi: 10.3389/fmicb.2022.828469 (PMC9006452; doi:10.3389/fmicb.2022.828469)

Supplementary Figure S1. Rarefaction curves of all samples for (A) observed ASVs, (B) Faith's phylogenetic diversity and (C) shannon diversity.

A

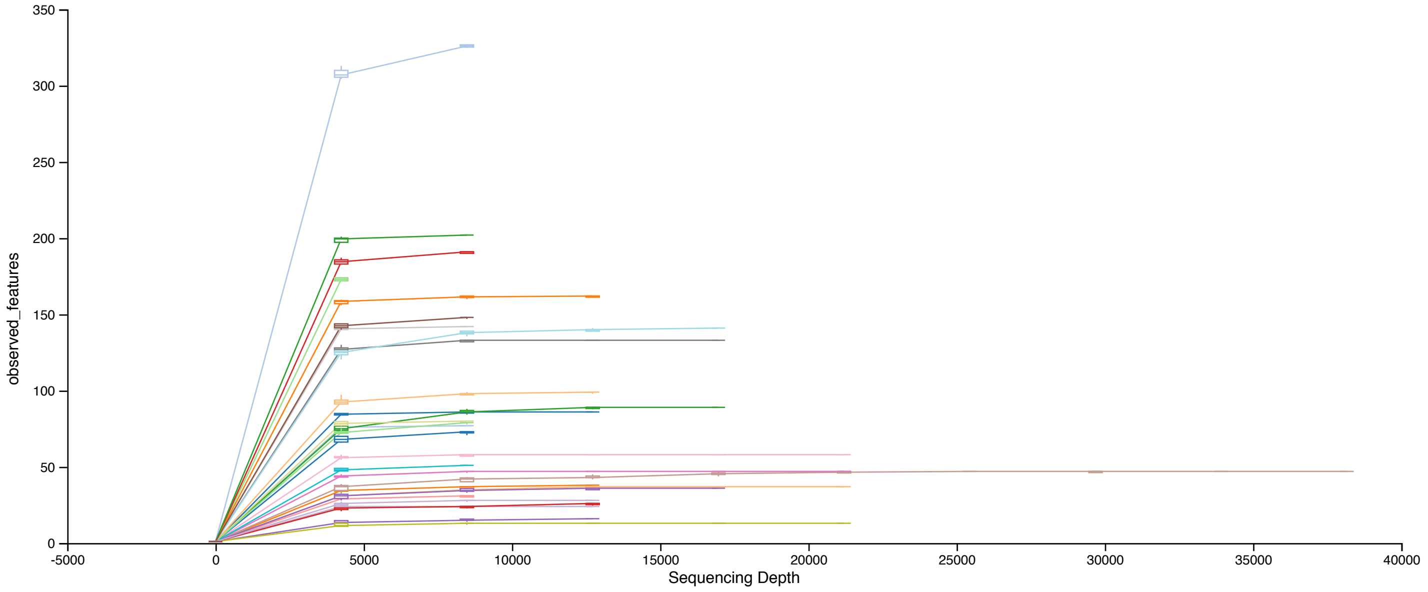

B

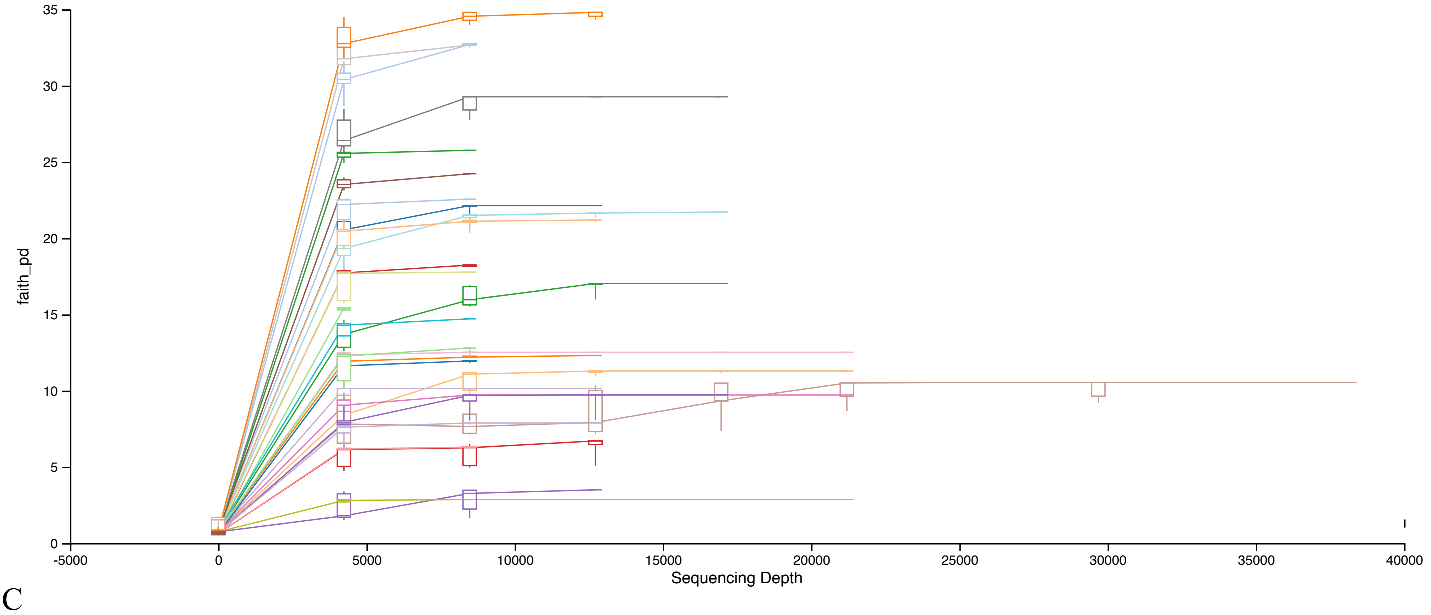

C

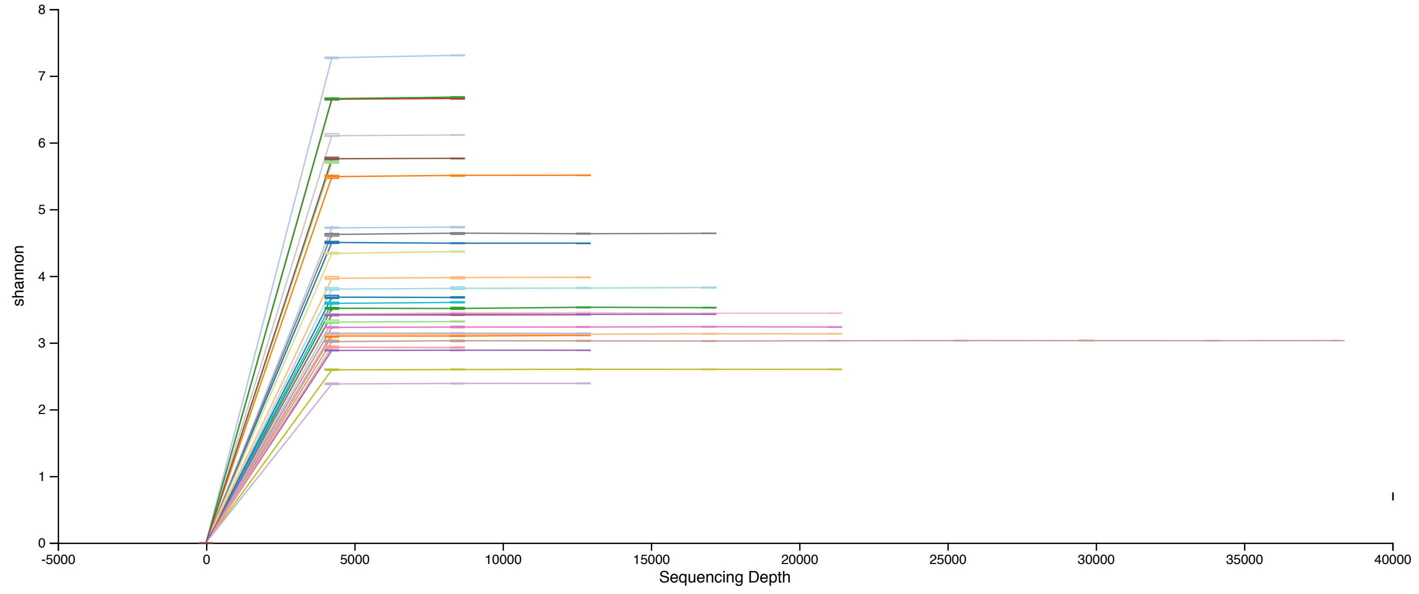

Supplement: Supplementary file 1 [file Data_Sheet_1.PDF]
