# Supplementary material for: Microbial Associations of Abyssal Gorgonians and Anemones (>4,000 m Depth) at the Clarion-Clipperton Fracture Zone
Source: Front Microbiol. 2022 Mar 30;13:828469. doi: 10.3389/fmicb.2022.828469 (PMC9006452; doi:10.3389/fmicb.2022.828469)
Supplement: Supplementary file 2 [file Data_Sheet_2.PDF]

Supplementary Figure S2. Similarity Percentage (SIMPER) analyses between sampling groups.

Groups Actinostolidae & Isididae

Average dissimilarity = 86.84

ASVs	Group Actinostolidae Av.Abund	Group Isididae Av.Abund	Av.Diss	Diss/SD	Contrib%	Cum.%
Hyphomicrobiaceae_ASV56	0.33	0.00	5.94	0.99	6.84	6.84
Spongiibacteraceae_ASV1	0.08	0.34	5.17	1.58	5.96	12.80
Spongiibacteraceae_ASV2	0.08	0.33	4.99	1.59	5.75	18.54
Parvibaculales_ASV33	0.27	0.03	4.56	1.14	5.25	23.79
Spongiibacteraceae_ASV3	0.07	0.30	4.47	1.58	5.14	28.93
Spongiibacteraceae_ASV5	0.07	0.28	4.27	1.60	4.92	33.85
Spongiibacteraceae_ASV4	0.07	0.28	4.25	1.60	4.90	38.75
Pelagibius_ASV60	0.25	0.00	4.25	1.11	4.89	43.64
Spongiibacteraceae_ASV6	0.06	0.27	4.12	1.59	4.75	48.38
Gammaproteobacteria_ASV59	0.21	0.00	3.91	0.68	4.50	52.89
Hyphomicrobiaceae_ASV75	0.21	0.00	3.68	1.17	4.24	57.12
Gammaproteobacteria_ASV43	0.13	0.02	2.66	0.53	3.06	60.18
Spongiibacteraceae_ASV13	0.03	0.15	2.30	1.41	2.64	62.82
Spongiibacteraceae_ASV14	0.03	0.14	2.16	1.37	2.49	65.31
Gammaproteobacteria_ASV39	0.08	0.02	1.49	0.57	1.72	67.04
Mycoplasma_ASV18	0.04	0.07	1.41	0.80	1.63	68.66
Mycoplasma_ASV16	0.04	0.06	1.30	0.82	1.50	70.16
Gammaproteobacteria_ASV23	0.00	0.08	1.23	0.52	1.41	71.57
Gammaproteobacteria_ASV24	0.00	0.08	1.22	0.56	1.40	72.98
Mycoplasma_ASV21	0.04	0.05	1.09	0.77	1.26	74.24
Actinomarinales_ASV83	0.06	0.00	1.08	0.73	1.24	75.48
Mycoplasma_ASV22	0.03	0.05	1.03	0.74	1.19	76.66
Ralstonia_ASV52	0.04	0.02	0.93	0.62	1.07	77.74
Ralstonia_ASV38	0.03	0.03	0.84	0.70	0.96	78.70
Pseudomonas_ASV31	0.03	0.03	0.80	0.78	0.92	79.62
Brevundimonas_ASV26	0.00	0.05	0.75	0.72	0.86	80.48
Acinetobacter_ASV28	0.00	0.05	0.71	0.68	0.81	81.29
Spongiibacteraceae_ASV7	0.00	0.03	0.63	0.24	0.73	82.02
Simkaniaceae_ASV25	0.00	0.03	0.58	0.45	0.67	82.69
Spongiibacteraceae_ASV8	0.00	0.03	0.58	0.24	0.67	83.35
Spongiibacteraceae_ASV10	0.00	0.02	0.55	0.24	0.64	83.99
Brevundimonas_ASV27	0.00	0.04	0.52	0.60	0.60	84.59
Spongiibacteraceae_ASV12	0.00	0.02	0.51	0.24	0.58	85.17
Spongiibacteraceae_ASV15	0.00	0.02	0.47	0.24	0.54	85.71
Actinomarinales_ASV55	0.02	0.02	0.46	0.52	0.53	86.24
Bacteria_ASV34	0.02	0.01	0.46	0.62	0.52	86.76
Bacteria_ASV42	0.02	0.02	0.46	0.53	0.52	87.28
Stenotrophomonas_ASV41	0.00	0.03	0.44	0.50	0.51	87.79
Vibrionaceae_ASV65	0.00	0.03	0.43	0.29	0.49	88.29

Entomoplasmatales_ASV32	0.00	0.03	0.43	0.36	0.49	88.78
Stenotrophomonas_ASV30	0.00	0.03	0.40	0.46	0.47	89.24
Synechococcales_ASV54	0.00	0.03	0.40	0.59	0.46	89.71
Actinomarinaceae_ASV45	0.01	0.02	0.40	0.62	0.46	90.17

Groups Actinostolidae & Primnoidae
Average dissimilarity = 99.11

ASVs	Group Actinostolidae Av.Abund	Group Primnoidae Av.Abund	Av.Diss	Diss/SD	Contrib%	Cum.%
Hyphomicrobiaceae_ASV56	0.33	0.00	6.52	0.99	6.58	6.58
Spongiibacteraceae_ASV7	0.00	0.33	6.03	2.06	6.08	12.66
Spongiibacteraceae_ASV8	0.00	0.31	5.79	2.08	5.84	18.50
Spongiibacteraceae_ASV10	0.00	0.28	5.20	2.08	5.25	23.75
Terasakiellaceae_ASV11	0.00	0.28	5.17	1.56	5.22	28.96
Parvibaculales_ASV33	0.27	0.00	5.11	1.12	5.16	34.12
Terasakiellaceae_ASV9	0.00	0.27	5.05	1.28	5.10	39.22
Spongiibacteraceae_ASV12	0.00	0.27	5.05	2.14	5.09	44.31
Spongiibacteraceae_ASV15	0.00	0.26	4.81	2.08	4.85	49.16
Pelagibius_ASV60	0.25	0.00	4.65	1.13	4.69	53.86
Terasakiellaceae_ASV17	0.00	0.24	4.41	1.28	4.44	58.30
Gammaproteobacteria_ASV59	0.21	0.00	4.30	0.67	4.34	62.64
Terasakiellaceae_ASV19	0.00	0.23	4.22	1.28	4.25	66.89
Terasakiellaceae_ASV20	0.00	0.22	4.10	1.28	4.13	71.03
Hyphomicrobiaceae_ASV75	0.21	0.00	4.03	1.19	4.07	75.09
Gammaproteobacteria_ASV43	0.13	0.00	2.75	0.49	2.77	77.87
Gammaproteobacteria_ASV39	0.08	0.00	1.39	0.49	1.40	79.27
Spongiibacteraceae_ASV1	0.08	0.02	1.39	0.62	1.40	80.67
Spongiibacteraceae_ASV2	0.08	0.02	1.34	0.62	1.35	82.02
Actinomarinales_ASV83	0.06	0.01	1.23	0.86	1.24	83.27
Spongiibacteraceae_ASV3	0.07	0.02	1.21	0.61	1.22	84.48
Spongiibacteraceae_ASV4	0.07	0.02	1.16	0.62	1.17	85.66
Spongiibacteraceae_ASV5	0.07	0.02	1.13	0.62	1.14	86.80
Spongiibacteraceae_ASV6	0.06	0.01	1.09	0.62	1.10	87.90
Ralstonia_ASV52	0.04	0.00	0.86	0.49	0.87	88.77
Endozoicomonas_ASV40	0.00	0.04	0.81	0.88	0.82	89.58
Alphaproteobacteia_ASV68	0.00	0.04	0.69	0.94	0.70	90.28

Groups Isididae & Primnoidae
Average dissimilarity = 93.50

ASVs	Group Isididae Av.Abund	Group Primnoidae Av.Abund	Av.Diss	Diss/SD	Contrib%	Cum.%
Spongiibacteraceae_ASV1	0.34	0.02	4.92	2.16	5.27	5.27
Spongiibacteraceae_ASV2	0.33	0.02	4.75	2.17	5.08	10.35

Spongiibacteraceae_ASV7	0.03	0.33	4.73	2.05	5.06	15.41
Spongiibacteraceae_ASV8	0.03	0.31	4.52	2.05	4.83	20.24
Spongiibacteraceae_ASV3	0.30	0.02	4.26	2.18	4.56	24.80
Terasakiellaceae_ASV11	0.00	0.28	4.18	1.59	4.47	29.27
Spongiibacteraceae_ASV10	0.02	0.28	4.09	2.07	4.37	33.64
Terasakiellaceae_ASV9	0.00	0.27	4.09	1.30	4.37	38.01
Spongiibacteraceae_ASV5	0.28	0.02	4.07	2.23	4.36	42.37
Spongiibacteraceae_ASV4	0.28	0.02	4.06	2.23	4.34	46.71
Spongiibacteraceae_ASV12	0.02	0.27	3.94	2.10	4.22	50.92
Spongiibacteraceae_ASV6	0.27	0.01	3.94	2.20	4.21	55.14
Spongiibacteraceae_ASV15	0.02	0.26	3.74	2.04	4.00	59.14
Terasakiellaceae_ASV17	0.00	0.24	3.56	1.30	3.81	62.95
Terasakiellaceae_ASV19	0.00	0.23	3.40	1.31	3.64	66.59
Terasakiellaceae_ASV20	0.00	0.22	3.31	1.30	3.54	70.13
Spongiibacteraceae_ASV13	0.15	0.00	2.22	1.70	2.38	72.51
Spongiibacteraceae_ASV14	0.14	0.00	2.09	1.63	2.24	74.75
Gammaproteobacteria_ASV23	0.08	0.00	1.11	0.53	1.18	75.93
Gammaproteobacteria_ASV24	0.08	0.00	1.10	0.57	1.17	77.10
Mycoplasma_ASV18	0.07	0.00	1.05	0.67	1.12	78.23
Mycoplasma_ASV16	0.06	0.00	0.92	0.66	0.98	79.21
Mycoplasma_ASV21	0.05	0.00	0.74	0.60	0.79	80.00
Brevundimonas_ASV26	0.05	0.01	0.70	0.80	0.75	80.75
Mycoplasma_ASV22	0.05	0.00	0.69	0.57	0.74	81.48
Endozoicomonas_ASV40	0.00	0.04	0.66	0.89	0.70	82.19
Acinetobacter_ASV28	0.05	0.00	0.64	0.68	0.68	82.87
Actinomarinaceae_ASV29	0.03	0.03	0.63	0.85	0.67	83.54
Brevundimonas_ASV27	0.04	0.01	0.58	0.75	0.62	84.16
Alphaproteobacteia_ASV68	0.00	0.04	0.56	0.96	0.60	84.76
_ASV38	0.03	0.01	0.54	0.56	0.58	85.34
Simkaniaceae_ASV25	0.03	0.00	0.52	0.45	0.55	85.89
Endozoicomonas_ASV53	0.00	0.03	0.49	0.69	0.52	86.41
Pseudomonas_ASV31	0.03	0.00	0.44	0.59	0.47	86.88
Parvibaculales_ASV33	0.03	0.00	0.44	0.37	0.47	87.35
Methyloligellaceae_ASV63	0.02	0.01	0.41	0.50	0.44	87.79
Stenotrophomonas_ASV41	0.03	0.00	0.40	0.50	0.43	88.22
Entomoplasmatales_ASV32	0.03	0.00	0.38	0.36	0.41	88.62
Vibrionaceae_ASV65	0.03	0.00	0.38	0.29	0.41	89.03
Stenotrophomona_ASV30	0.03	0.00	0.37	0.46	0.39	89.42
Cyanobiaceae_ASV54	0.03	0.00	0.36	0.60	0.39	89.81
Sphingomonas_ASV46	0.03	0.00	0.36	0.50	0.38	90.19
